# What QLQ-C15-PAL Symptoms Matter Most for Overall Quality of Life in Patients With Advanced Cancer?

**DOI:** 10.4021/wjon330w

**Published:** 2011-08-24

**Authors:** Amanda Caissie, Shaelyn Culleton, Janet Nguyen, Liying Zhang, Liang Zeng, Lori Holden, Kristopher Dennis, Esther Chan, Florencia Jon, May Tsao, Cyril Danjoux, Arjun Sahgal, Elizabeth Barnes, Kaitlin Koo, Edward Chow

**Affiliations:** a Rapid Response Radiotherapy Program, Odette Cancer Centre, Sunnybrook Health Sciences Centre, University of Toronto, Toronto, Ontario, Canada

**Keywords:** QLQ-C15-PAL, Quality of life, Bone metastases, Brain metastases, Advanced lung cancer, Radiotherapy

## Abstract

**Background:**

Few studies have evaluated the QLQ-C15-PAL health-related quality of life (QOL) questionnaire, an abbreviated version of the QLQ-C30 questionnaire that was designed specifically for patients with advanced cancer. The present study assessed whether certain symptoms or functional domains from the QLQ-C15-PAL predicted overall QOL when rated prior to palliative radiation treatment (RT).

**Patients and Methods:**

Patients attending an outpatient palliative radiotherapy clinic completed QLQ-C15-PAL questionnaires prior to palliative RT for bone, brain or lung disease. Pearson correlations were computed between the QLQ-C15-PAL functional/symptom scores and overall QOL scores. Multiple linear regressions were used to evaluate the relative importance of functional/symptom scales in association with overall QOL.

**Results:**

Data from 369 patients were analyzed. The QLQ-C15-PAL domains of physical and emotional functioning, pain, and appetite loss were significant predictors of overall QOL in these patients with advanced cancer. Appetite loss was the only significant independent predictor of overall QOL in the subgroup of patients with advanced lung cancer (n = 29). Both appetite loss and emotional functioning were independently predictive of overall QOL in patients with bone metastases (n = 190). In patients with brain metastases (n = 150), independent predictors of overall QOL included physical and emotional functioning as well as fatigue.

**Conclusion:**

The QLQ-C15-PAL domains of physical and emotional functioning, pain and appetite loss were significant predictors of overall QOL in this cohort of patients with advanced cancer. Different functional and symptom scales predicted overall QOL in patients with bone metastases, brain metastases or advanced lung cancer.

## Introduction

Traditional endpoints of overall survival are no longer the primary endpoint of most palliative oncology clinical trials and have been replaced with more patient-specific determinants measuring symptom control and quality of life (QOL) [1]. Assessing treatment effect on QOL is particularly challenging given that there has yet to be a consensus with respect to a unified definition of QOL, which generally includes factors beyond health in the context of an individual’s culture and value system [2]. As a component of overall QOL, health-related quality of life (HRQOL) is a subjective multidimensional construct that includes physical and psychosocial factors related to patient health care [3]. To address the need for standardized assessment of HRQOL in oncology patients, various HRQOL questionnaires have been developed including the well established QLQ-C30 questionnaire developed by the European Organization for Research and Treatment of Cancer (EORTC) [4].

In studies that have assessed the prevalence of distressful symptoms in patients with advanced cancer, up to 85% of patients have reported at least one moderate to severe symptom [5]. With such a significant symptom burden, it becomes important to identify which symptoms matter most to the patient in terms of overall QOL, so that palliative treatments and supportive care may be tailored accordingly. In the lung cancer literature, this concept has been explored using the EORTC QLQ-C30 to investigate the relative importance of various HRQOL symptoms and functional domains in predicting overall QOL [6, 7].

An abbreviated version of the QLQ-C30, known as the QLQ-C15-PAL, has been developed to decrease the burden of the longer more time-consuming parent questionnaire for patients with advanced cancer [8]. Although abbreviated, the QLQ-C15-PAL still provides a single, convenient platform to measure HRQOL symptoms and functional domains in addition to overall globally-rated QOL. However, few studies have actually integrated or evaluated the QLQ-C15-PAL in its intended target population. Using the QLQ-C15-PAL in a cohort of patients with advanced cancer, the present study assessed the relative importance of different functional/symptom domains in predicting overall QOL ratings prior to palliative radiation treatment (RT) of symptomatic bone, brain or lung lesions.

## Patients and Methods

### Patients

The Rapid Response Radiotherapy Program (RRRP) is a rapid-access outpatient palliative RT clinic running daily in the Odette Cancer Centre at the Sunnybrook Health Sciences Centre, Toronto, Ontario Canada. All research was therefore conducted following approval from the Sunnybrook Health Science Centre research ethics board. This study included patients seen in RRRP consultation between October 2007 and July 2010, with completed QLQ-C15-PAL questionnaires prior to palliative RT for bone metastases, brain metastases or advanced lung cancer. The term advanced lung cancer will be used throughout the manuscript referring to patients who were seen in consultation for palliative RT of primary lung cancer or lung metastasis. Baseline patient data collected included age, Karnofsky performance status (KPS), gender, primary cancer site and presence of other metastases, including visceral or bone metastases outside of the RT site.

### The QLQ-C15-PAL

Prior to RT, general HRQOL and overall QOL was assessed using the QLQ-C15-PAL. This QOL measurement tool consists of 15 questions; two multi-item functional scales (physical and emotional functioning), two multi-item symptom scales (fatigue and pain) along with five single item symptom scales (nausea/vomiting, dyspnoea, insomnia, appetite loss, constipation) and one final question referring to overall QOL. The physical functioning scale is based on three questions that ask about walking, activities of daily living and time spent in bed or in a chair. The emotional functioning scale is based on two questions that ask about feeling tense or depressed. Patients rated each question/item on a numeric scale from 1 (not at all) to 4 (very much), with the exception of global QOL which was rated from 1 (very poor) to 7 (excellent). The EORTC QLQ-C30 scoring manual [9] was used to generate the QLQ-C15-PAL scores (0-100) for the unchanged pain scale and the four single items unchanged from the QLQ-C30 (dyspnoea, insomnia, appetite loss, constipation). Scores (0-100) for the remaining scales were generated using the QLQ-C15-PAL scoring addendum available from the EORTC Quality of Life Unit.

### Statistical analysis

Results were initially analyzed for the entire group of patients with advanced cancer. Demographics were expressed as proportions for categorical variables but means, standard deviations (SD), inter-quartiles, medians and ranges (minimum to maximum) for continuous variables. Pearson correlations were computed between all functional/symptom scales and the overall QOL scale. The strength of the relationship was indicated by the correlation coefficient (r). The larger the correlation, the stronger the relationship. The following rules were used for categorizing r: ≤ 0.50 very low; 0.51-0.79 low; 0.80-0.89 moderate, ≥ 0.90 high. The significance of the relationship was expressed in probability levels of p-value. The smaller the p-value, the more significant the relationship. When sample size is large, a small r-value may be significant but with a weaker relationship [10].

A multiple linear regression analysis was then used to evaluate the relative importance of different functional/symptoms scales in predicting overall QOL. Two separate models were computed. In the first model, the 2 functional scale variables were entered simultaneously with the regression equation. In the second model, the 7 symptom scale variables were entered simultaneously with the regression equation. Finally, variables found to be statistically significant in previous regression analyses were entered together as independent variables in predicting overall QOL. The above process of statistical analysis was performed for each subgroup of patients with symptomatic bone, brain and lung disease. Two-tailed p-values less than 0.05 were considered statistically significant. All analyses were conducted by Statistical Analysis Software (SAS for Windows, version 9.2).

## Results

### QOL analysis in all patients

A total of 369 patients with advanced cancer completed the QLQ-C15-PAL questionnaires prior to palliative RT for bone (190 patients), brain (150 patients) or lung disease (29 patients). Baseline patient characteristics are presented in Table 1.

**Table 1:** Patient Characteristics (n = 369)

Characteristics	Value	
Age (years)		
n	369	
Mean ± SD	65 ± 12	
Inter-quartiles	58 – 75	
Median (range)	66 (22 – 89)	
KPS		
n	362	
Mean ± SD	67 ± 15	
Inter-quartiles	60 – 80	
Median (range)	70 (30 – 100)	
Gender		
Male	199 (54%)	
Female	170 (46%)	
Primary cancer site		
Lung	145 (39%)	
Breast	73 (20%)	
Prostate	64 (17%)	
Renal Cell	28 (8%)	
Colorectal	18 (5%)	
Unknown	8 (2%)	
Others	33 (9%)	

Pearson correlations in all patients showed that all functional and symptom scales were significantly correlated with overall QOL (Table 2), although the small r-value may have indicated very low or low relationships. In the initial multiple linear regression analysis (Table 3), both physical and emotional functioning significantly predicted the overall QOL (p < 0.0001). Fatigue (p < 0.0001), pain (p = 0.0009) and appetite loss (p = 0.0041) were significant symptoms predictive of overall QOL. The functional and symptom scales accounted for 35% and 28% of the variance in overall QOL, respectively. Positive coefficients of the two functional scales indicated that higher functional scores (better physical and emotional functioning) predicted higher overall QOL. Negative coefficients of the three significant symptom scales indicated that higher symptom scales (more symptom burden) predicted lower overall QOL.

**Table 2:** Pearson Correlations Between all Functional/Symptom Scale Variables and Overall QOL in All Patients With Advanced Cancer

Variable	Correlation (r)	p-value^a^	n
Physical Functioning	0.43	< 0.0001	352
Emotional Functioning	0.53	< 0.0001	357
Fatigue	-0.46	< 0.0001	361
Nausea / Vomiting	-0.28	< 0.0001	361
Pain	-0.41	< 0.0001	362
Dyspnoea	-0.19	0.0004	361
Insomnia	-0.27	< 0.0001	362
Appetite loss	-0.39	< 0.0001	361
Constipation	-0.24	< 0.0001	362

a. All p-values are less than 0.05 and considered statistically significant.

**Table 3:** Multiple Linear Regression Analysis of All Functional/Symptom Scale Variables With Overall QOL^a^ in All Patients With Advanced Cancer

Variable	Coefficient	Standardized Coefficient	t	p-value
Functional scales				
Physical Functioning	0.27	0.29	6.32	< 0.0001*
Emotional Functioning	0.45	0.44	9.55	< 0.0001*
	Intercept = 7.30, multiple R = 0.60, multiple R^2^ = 0.35, adjusted R^2^ = 0.35			
Symptom scales				
Fatigue	-0.23	-0.24	-4.09	< 0.0001*
Nausea / Vomiting	-0.02	-0.02	-0.40	0.69
Pain	-0.14	-0.19	-3.35	0.0009*
Dyspnoea	-0.03	-0.03	-0.57	0.57
Insomnia	-0.05	-0.07	-1.42	0.16
Appetite loss	-0.12	-0.16	-2.89	0.0041*
Constipation	-0.03	-0.03	-0.69	0.49
	Intercept = 78.56, multiple R = 0.54, multiple R^2^ = 0.29, adjusted R^2^ = 0.28			

a.Overall QOL was considered the outcome. In the first model, the 2 functional scales were entered simultaneously in the regression equation, while in the second model, the 7 symptom scales were entered simultaneously. * P-values less than 0.05 are considered statistically significant.

When the significant functional scale and significant symptom scale variables were put into one model (Table 4), both functional scale variables (p < 0.0001 for emotional; p = 0.0026 for physical) along with symptoms of pain and appetite loss (p = 0.025 for both) were significantly related to overall QOL. The functional and symptom scale variables together accounted for 39% of the variance in overall QOL.

**Table 4:** Multiple Linear Regression Analysis With Physical and Emotional Functioning, Pain and Appetite Loss as Predictors of Overall QOL^a^ in all Patients With Advanced Cancer

Variable	Coefficient	Standardized Coefficient	t	p-value
Functional scales				
Physical Functioning	0.15	0.16	3.04	0.0026*
Emotional Functioning	0.37	0.36	7.56	< 0.0001*
Symptom scales				
Fatigue	-0.10	-0.11	-1.89	0.06
Pain	-0.09	-0.12	-2.25	0.0251*
Appetite loss	-0.09	-0.11	-2.25	0.0253*
	Intercept = 31.52, multiple R = 0.64, multiple R^2^ = 0.40, adjusted R^2^ = 0.39			

a. Overall QOL was considered the outcome. Variables found to be statistically significant for all patients (Table 2) were entered concurrently as independent variables in predicting overall QOL. * P-values less than 0.05 are considered statistically significant.

### QOL analysis in patient subgroups

Baseline characteristics are presented in Table 5 for the three subgroups of patients with advanced cancer of bone, brain and lung.

**Table 5:** Patient Characteristics of the Three Patient Subgroups

Characteristics	Bone Metastases (n = 190)	Brain Metastases (n = 150)	Advanced Lung Cancer (n = 29)
Age (years)			
n	190	150	29
Mean ± SD	67 ± 13	63 ± 11	68 ± 12
Inter-quartiles	59 – 77	56 – 71	58 – 78
Median (range)	68 (26–89)	64 (22–86)	70 (38–85)
KPS			
n	184	149	29
Mean ± SD	67 ± 14	74 ± 15	67 ± 16
Inter-quartiles	60 – 80	60 – 90	60 – 80
Median (range)	70 (30–100)	80 (30–100)	70 (30–90)
Gender			
Male	116 (61%)	65 (43%)	18 (62%)
Female	74 (39%)	85 (57%)	11 (38%)
Primary cancer site			
Lung	41 (22%)	80 (53%)	24 (83%)
Breast	42 (22%)	30 (20%)	1 (4%)
Prostate	63 (33%)	1 (1%)	0 (0.0%)
Renal Cell	18 (10%)	9 (6%)	1 (4%)
Colorectal	6 (3%)	9 (6%)	3 (10%)
Unknown	4 (2%)	4 (3%)	0 (0.0%)
Others	16 (8%)	17 (11%)	0 (0.0%)
Other metastases			
No	146 (77%)	79 (53%)	9 (31%)
Yes	44 (23%)	71 (47%)	20 (69%)

### Bone metastases subgroup

With the exception of dyspnoea, all functional and symptom scale variables were significantly correlated with overall QOL according to Pearson correlations (Table 6), although the small r-value indicated very low or low relationships. Through regression analysis, both physical (p = 0.0038) and emotional (p < 0.0001) functional scales as well as the symptom scales of pain (p = 0.003) and appetite loss (p = 0.007) were initially identified as significant predictors of overall QOL. The functional and symptom scales accounted for 32% and 21% of the variance in overall QOL, respectively. When significant variables were put into one model, only emotional functioning (p < 0.0001) and appetite loss (p = 0.024) were significantly predictive of overall QOL (Table 7). The functional and symptom scales together explained 35% of the variance in overall QOL.

**Table 6:** Pearson Correlations Between all Functional/Symptom Scale Variables and Overall QOL in the Three Patient Subgroups

Variable	Bone Metastases			Brain Metastases			Advanced Lung Cancer		
	Correlation (r)	p-value^a^	n	Correlation (r)	p-value^a^	n	Correlation (r)	p-value^a^	n
Physical Functioning	0.33	< 0.0001*	181	0.44	< 0.0001*	145	0.58	0.0020*	26
Emotional Functioning	0.54	< 0.0001*	184	0.50	< 0.0001*	146	0.71	< 0.0001*	27
Fatigue	-0.36	< 0.0001*	185	-0.51	< 0.0001*	148	-0.48	0.0096*	28
Nausea / Vomiting	-0.26	0.0003*	185	-0.22	0.0066*	148	-0.41	0.0293*	28
Pain	-0.40	< 0.0001*	185	-0.34	< 0.0001*	149	-0.27	0.1585	28
Dyspnoea	-0.12	0.1025	185	-0.17	0.0363*	149	-0.48	0.0121*	27
Insomnia	-0.18	0.0122*	185	-0.28	0.0006*	149	-0.42	0.0263*	28
Appetite loss	-0.39	< 0.0001*	185	-0.20	0.0141*	149	-0.77	< 0.0001*	27
Constipation	-0.17	0.0211*	185	-0.22	0.0081*	149	-0.28	0.1532	28

a. * P-values less than 0.05 are considered statistically significant.

**Table 7:** Multiple Linear Regression Analysis With Emotional Functioning and Appetite Loss Associated With Overall QOL^a^ in Patients With Bone Metastases

Variable	Coefficient	Standardized Coefficient	t	p-value
Functional scales				
Physical Functioning	0.08	0.09	1.22	0.22
Emotional Functioning	0.36	0.41	6.06	< 0.0001*
Symptom scales				
Pain	-0.11	-0.13	-1.74	0.08
Appetite loss	-0.11	-0.16	-2.27	0.0242*
	Intercept = 31.58, multiple R = 0.60, multiple R^2^ = 0.36, adjusted R^2^ = 0.35			

a. Overall QOL was considered the outcome. Initial modeling identified significant functional/symptom scales that were then entered concurrently as independent variables. * P-values less than 0.05 are considered statistically significant.

### Brain metastases subgroup

Pearson correlations showed that all functional and symptom scales were significantly correlated with overall QOL (Table 6). Through regression analysis, both physical and emotional functional scales (p < 0.0001 for both) as well as the symptom scale of fatigue (p < 0.0001) were initially identified as significant predictors of overall QOL. Both functional scales and fatigue accounted for 31% and 24% of the variance in overall QOL, respectively. All three initially significant variables were predictive of overall QOL when put into one model (p < 0.0001 for emotional; p = 0.02 for physical; p = 0.001 for fatigue) (Table 8). The functional and symptom scales together explained 37% of the variance in overall QOL.

**Table 8:** Multiple Linear Regression Analysis With Physical and Emotional Functioning and Fatigue Associated With Overall QOL^a^ in Patients With Brain Metastases

Variable	Coefficient	Standardized Coefficient	t	p-value
Functional scales				
Physical Functioning	0.18	0.19	2.36	0.0198*
Emotional Functioning	0.38	0.33	4.57	< 0.0001*
Symptom scales				
Fatigue	-0.28	-0.28	-3.36	0.001*
	Intercept = 33.18, multiple R = 0.62, multiple R^2^ = 0.3, adjusted R^2^ = 0.372			

a. Overall QOL was considered the outcome. Initial modeling identified significant functional/symptom scales that were then entered concurrently as independent variables. * P-values less than 0.05 are considered statistically significant.

### Advanced lung cancer subgroup

With the exception of pain and constipation, all functional and symptom scales were significantly correlated with overall QOL according to Pearson correlations (Table 6). Through regression analysis, both physical (p = 0.026) and emotional (p = 0.001) functional scales as well as the symptom scale of appetite loss (p = 0.001) were initially identified as significant predictors of overall QOL. Both functional scales and appetite loss accounted for 54% and 58% of the variance in overall QOL, respectively. Only appetite loss (p = 0.035) was found to be predictive of overall QOL, when initially significant variables were put into one model (Table 9). The functional and symptom scales together explained 61% of the variance in overall QOL.

**Table 9:** Multiple Linear Regression Analysis With Physical and Emotional Functioning and Fatigue Associated With Overall QOL^a^ in Patients With Advanced Lung Cancer

Variable	Coefficient	Standardized Coefficient	t	p-value
Functional scales				
Physical Functioning	0.19	0.19	1.23	0.23
Emotional Functioning	0.43	0.33	1.98	0.06
Symptom scales				
Appetite loss	-0.34	-0.43	-2.26	0.0347*
	Intercept = 19.43, multiple R = 0.81, multiple R^2^ = 0.66, adjusted R^2^ = 0.61			

a. Overall QOL was considered the outcome. Initial modeling identified significant functional/symptom scales that were then entered concurrently as independent variables. * P-values less than 0.05 are considered statistically significant.

## Discussion

In the palliative setting when facing treatment decisions for patients with poor prognosis, symptom control and QOL become arguably the most important goals of care rather than traditional oncologic endpoints of survival [1]. While objective endpoints such as overall survival are easily measured, standardization of subjective endpoints is possible with various HRQOL instruments such as the cancer-specific EORTC QLQ-C30 questionnaire [4] allowing for reproducibility across studies. Although the QLQ-C30 is one of the most frequently used measures of HRQOL in oncology clinical trials, it is lengthy. Certain studies have had issues with patient completion of questionnaires, particularly when combining the QLQ-C30 with disease-specific questionnaires such as the brain cancer-specific BN20 tool [11, 12]. Since the QLQ-C30, progress has been made in the field of HRQOL research with the development of an abbreviated QLQ-C15-PAL for patients with advanced cancer [8].

It has previously been proposed that HRQOL is antecedent to overall QOL and that it is important to differentiate between the two [6]. Using the QLQ-C30, fatigue and social functioning have been identified as independent predictors of overall QOL in 98 oncology patients with an estimated life expectancy of 1-6 months [13]. The QLQ-C15-PAL is an abbreviated HRQOL questionnaire consisting of two multi-item functional scales (physical and emotional functioning) along with seven symptom scales. In contrast to the QLQ-C30, there is only one question referring to overall QOL in the QLQ-C15-PAL. There have been several editorials discussing the use of QLQ-C15-PAL [14, 15] along with several papers discussing the planned use of QLQ-C15-PAL in upcoming studies [16-18]. However, to our knowledge only two other studies to date have assessed QOL using the QLQ-C15-PAL **s**ince its development in 2006 [19, 20]. Suarez-Del-Real et al. validated the Mexican-Spanish version of the QLQ-C15-PAL questionnaire for the evaluation of HRQOL in patients with advanced cancer receiving palliative care [19]. In a study of patients with brain metastases, Steinmann et al. found better compliance and practicality with the abbreviated QLQ-C15-PAL as compared to the QLQ-C30, and the group reported current use of the QLQ-C15-PAL in their ongoing larger scale study [20]. In the pilot study, the QLQ-C15-PAL identified pre-RT symptoms ranging from little nausea to more prominent fatigue and insomnia [20].

To our knowledge, this is the first study using the QLQ-C15-PAL to explore dimensions of HRQOL which may predict overall QOL in patients with advanced cancer. In the initial analysis of all 369 patients with advanced cancer, multiple regressions identified both physical and emotional functional scales as well as pain and appetite loss symptoms as significant predictors of overall QOL. This initial analysis included a broad group of patients with respect to their individual characteristics and tumor types. As the symptom burden is often very different depending on the disease site, sub-group analysis was performed. It is acknowledged that not all patients fit specifically into discrete categories as patients with advanced cancer often have multiple sites of disease. In this study, other metastases were present in 23%, 47% and 69% of patients with bone metastases, brain metastases and advanced lung cancer, respectively. By classifying patients according to the main reason for referral, this study has shown that different functional and symptom scales have varying significance to overall QOL in specific subpopulations of patients with advanced cancer.

In the relatively small group of patients with advanced lung cancer (n = 29), appetite loss was the only significant predictor of overall QOL. Emotional and physical functioning were initially significant but were not identified as independent predictors in the final model. These results are in contrast to a previously published study in lung cancer, identifying emotional functioning and fatigue as independent predictors of overall QOL, using the core QLQ-C30 along with the lung cancer-specific QLQ-LC13 questionnaire [7]. The authors concluded that emotional concerns may be of more importance to this lung cancer population with a potentially life-threatening disease as compared to a population of patients with chronic lung disease excluding cancer, in which physical functioning had been found to predict overall QOL [21]. Likewise, it may be proposed that overall QOL may be driven by different factors in patients with advanced versus localized and potentially curable cancer. The present study focused on patients with advanced lung cancer. Therefore, results may not reflect those found by Ostlund et al. [7] who reported on a more heterogeneous patient population, in which only 29 of 52 patients were identified as having stage 3b or 4 lung cancer. The present study’s interpretation of results may also be limited by the small sample size of patients with advanced lung cancer.

Our cohort consisted of a large number of patients with bone metastases (n = 190). In this patient population, emotional functioning and appetite loss were found to be predictive of overall QOL. Although pain is often the primary reason for referral of patients with bone metastases to a palliative RT clinic, pain was not predictive of overall QOL in this assessment. These findings are consistent with previous work using a variety of questionnaires, identifying psychosocial issues such as depression, and not pain, to be most predictive of overall QOL in patients with bone metastases [22]. While it has been shown that health care providers consider symptom issues such as pain to be more important for their patients with bone metastases, patients themselves emphasize the importance of psychosocial issues [23].

In the present study’s assessment of patients with brain metastases, predictors of overall QOL included both emotional and physical functioning as well as fatigue. The emotional burden of a brain metastases diagnosis may be multi-factorial, including the extremely poor prognosis associated with it and the current or anticipated cognitive/physical impairments which lead to loss of independence [24]. As most patients with brain metastases are taking dexamethasone during RT, it is not surprising that nausea and vomiting were not predictors of overall QOL. Steroid use may also explain why loss of appetite was predictive of overall QOL in patients with bone metastases or advanced lung cancer but not brain metastases.

The data from this study, like those from studies using the QLQ-C30 [7, 13], does not support a previous proposal that symptoms affect patient functioning but not overall QOL directly [25]. The current study has in fact highlighted the relative importance that patients place on certain symptoms such as appetite loss or fatigue, which are extremely common as cancer progresses [26] yet often neglected with respect to assessment or management [27, 28]. One recent study of advanced cancer patients has shown a statistically significant incremental decline in patient satisfaction with QOL for every 10 unit increase in appetite loss on the QLQ-C30 [29].

In conclusion, symptom control and QOL preservation are crucial goals in oncology. Standardized assessments of such subjective endpoints are now possible through a wide range of tools such as the QLQ-C15-PAL. Using the QLQ-C15-PAL, this study has shown that different functional and symptom scale variables predict overall QOL in patients with bone metastases, brain metastases or advanced lung cancer prior to palliative RT. It is acknowledged that such an assessment may be affected by various features including the assessment tool used and the patient population under investigation. While this is a study of patients referred for RT, results may vary in those patients receiving different treatment modalities. Future studies may wish to investigate whether the treatments themselves subsequently affect overall QOL and predictors thereof. Simply assessing the single variable of overall QOL may be important when an outcome variable is required or a global impression of QOL is desired [30]. On the other hand, multi-variable assessment, as is possible with the QLQ-C15-PAL, provides a broader, richer view of the situation [31]. In the present study, the QLQ-C15-PAL was used in its intended patient population with advanced cancer to identify HRQOL functional domains and symptoms which matter most for overall QOL.
